# The Causation of Disease.—II


**Published:** 1889-05-25

**Authors:** 


### The Causation of Disease.—II.*

In previous observations on this very interesting, able, and learned work, the conclusion of the author was noted, that all disease depends upon peculiarity or abnormality of either cell structure or cell environment, or of both. "Each cell is fixed by a connecting tissue substance, and each dwells in a tiny chamber, where it lies bathed in a nutrient fluid . . . from which it derives its food, and to which it returns its refuse." All disease is ultimately an improper interaction between the cell and its nutrient fluid. There are several ways and means by which cell environments may be influenced and morbidly modified. All the tissues of the body are knit together into one physiological whole, so that if disease originates in one tissue it is apt to implicate others. For example, a slight defect in one of the heart-valves may ultimately lead to an altered environment of every cell in the body. Again, if a digestive juice be faulty, blood plasma will be imperfectly formed, and thus every cell in the body will suffer for the error in one limited patch of tissue. It is thus almost impossible to have one portion of the system faulty without throwing the whole machine out of gear; and the question therefore arises how far it is possible to have a purely local disease? On this point the author observes: "Supposing a condition such as atheroma, or fatty tumour, to be strictly local; and supposing, moreover, that it causes no trouble or nconvenience, the question arises, are we justified in regarding it as disease?" And the author answers this question by declaring we are not justified in regarding it as actual disease. If it contain within it the germ of some future mischief, if a fatty tumour be destined one day to press upon some nerve and cause pain, or if an atheromatous patch be but the germ of a future aneurism, then we have examples of potential, but not actual diseases. Now, in our opinion, this is not only incorrect, but not in accord with Dr. Campbell's own views. Disease, he states, consists of abnormal interaction between the cell and its environment; in other words, in change of structure. Atheroma and fatty tumour are abnormal change of structure, and therefore must constitute actual disease, even although the change is microscopically minute.

We now may apply the foregoing to practical purposes. If an individual has no tendency to disease, if his structure is perfect, and if he passes life under perfect cell environment, the vital processes go on in a normal manner, and there will be health. To a healthy body evil will only come from environment. It therefore follows that through environment alone can beneficial action be exerted on cells. This is accomplished by enforcing the principles of hygiene, and by administering drugs. The author suggestively adds, "the wise physician well knows upon which he places the most faith."

The second aspect under which causation of disease is to be studied is the individual structure of the cells themselves, and the author correctly holds that whatever affects structure may play a part in the production of disease. The proposition is laid down that "the structure of any individual depends chiefly on the structure of his more immediate progenitors." With this we do not entirely agree, for it is a fact in heredity that a child may often show a resemblance to a very distant ancestor, which none of his progenitors may have evidenced for generations. But ignoring this, Dr. Campbell asserts "as each of the many species of plant and animal gives origin to an offspring like itself, it is evident that heredity is the great power which determines the particular structure of an individual." And the author goes on to remark that "if heredity were unhampered there would be complete identity among the male offspring born of the same parents on the one hand, and among the female on the other; but this identity is destroyed by the diversity of environment." The latter conclusion may not be questioned, for, as Haekel remarks, every organism is dependent on the whole of its external surroundings and changed by their alteration. But were heredity unhampered, it may be doubted if there would be complete identity among offspring. The broad principle may be admitted, that a human family, tribe, or nation is liable in the course of generations to be either advanced from a mean form to a higher one, or degraded from a higher to a lower, by the influence of the physical conditions in which it lives. But whether ascending or descending, there will be infinite individual peculiarities and dissimilarities. Such dissimilarities, moreover, may appear in one generation, disappear in the next, and reappear in the third. Sexual reproduction consists in the union of two pieces of protoplasm, derived from separate organisations. Such atoms of protoplasm must contain a something which afterwards increases into a powerful agency for good or evil. Protoplasm, simple or nucleated, must be regarded as the basis of life, although not as life itself. All organisms, vegetable as well as animal, commence with a simple cell, of which it is impossible to tell to what form it is destined to advance. There is no essential difference between the vertebral column of the early embryo of man and that of an embryo fish. Sex is also a matter of development. All beings are at one stage of their embryotic process neuter. No environment will form the future living being or differentiate the sex. Therefore, although environment will do much, its absence would not entail a sequence of complete identity of offspring.

In external and internal environment the potency of rhythm is marked, or, as we should prefer to term it, "periodicity." Cycle, rhythm, or periodicity characterises all creation. Day and night, seasons, tides, phases of the moon, vital phenomena of various kinds, occur and recur periodically. All these are influences, or, to use the author's term, environment, acting powerfully on the human frame. The author quotes Dr. Creighton Brown, who observes: "It is certain that the seasons have a powerful hold on the human organism. There is a gain of body weight in winter and a loss in summer, and vital statistics show that each season has diseases which may be called peculiarly its own." We do not, however, require the arguments the author has advanced to demonstrate periodicity in man. It is daily exemplified by the rhythm of sleep, by the occurrence of hunger, by menstruation, and in disease by the periodicity of ague, of remittent fever, of migrane, and of various other maladies generally spoken of under the unmeaning term malarious. The cause of rhythm or periodicity in man can only be referred to man being under a dominant law of nature. This is, at least, as satisfactory as Darwin's suggestion. Alluding to the influence of the lunar rhythm on man, Darwin pointed out that it distinctly influences many aquatic animals through its action on the tides, and he hinted that man may have faintly inherited the rhythmical impress from some far-off aquatic ancestors. In our opinion, however, it does not much matter how it originated in man. The truth we have to deal with is its presence; and as regards the diagnosis and treatment of disease, it is well that it is so. The very fact of a malady evidencing periodicity generally stamps it as belonging to the class known as malarious, and indicates that remedies known as antiperiodics, such as quinine and arsenic, should form at least part of the treatment.

But rhythmical habits may be acquired, and such habits may

* "An Exposition of the Ultimate Factors which induce it." By Harry Campbell, M.D., B.S., London, Member of the Royal College of Physicians; Assistant-Physician and Pathologist to the North-West London Hospital. H. K. Lewis, 136, Gower-street. 1889.

interfere with health. A man may educate himself to feel sleepy at a particular hour or to wake at a specified time. He may educate himself to visit the closet at a certain hour, and so exacting is this acquired rhythm that often, if the individual does not obey the call at the proper time, the delay prevents defecation afterwards. We can further educate ourselves to feel hungry at particular hours. A man accustomed to smoke at a particular hour will experience the desire to indulge at that hour. And so on might be noted many other rhythmical habits. This, again, is satisfactory rather than the reverse, for habits may often be enlisted by the physician as one of his aids in the treatment of disease.

